# Angiotensinogen rs5050 germline genetic variant as potential biomarker of poor prognosis in astrocytoma

**DOI:** 10.1371/journal.pone.0206590

**Published:** 2018-11-01

**Authors:** Alexander Perdomo-Pantoja, Sonia Iliana Mejía-Pérez, Nancy Reynoso-Noverón, Liliana Gómez-Flores-Ramos, Ernesto Soto-Reyes, Thalía Estefania Sánchez-Correa, Lissania Guerra-Calderas, Clementina Castro-Hernandez, Silvia Vidal-Millán, José Sánchez-Corona, Lucia Taja-Chayeb, Olga Gutiérrez, Bernardo Cacho-Diaz, Rosa Maria Alvarez-Gomez, Juan Luis Gómez-Amador, Patricia Ostrosky-Wegman, Teresa Corona, Luis Alonso Herrera-Montalvo, Talia Wegman-Ostrosky

**Affiliations:** 1 Department of Neurosurgery, Johns Hopkins University School of Medicine, Baltimore, United States of America; 2 Departamento de Neurocirugía, Instituto Nacional de Neurología y Neurocirugía, "Manuel Velasco Suarez", Mexico City, Mexico; 3 Dirección de Investigación, Instituto Nacional de Cancerología, Mexico City, Mexico; 4 Unidad de Investigación Biomédica en Cáncer, Instituto de Investigaciones Biomédicas, UNAM-INCAN, Mexico City, Mexico; 5 Centro de Investigación Biomédica de Occidente, IMSS, Guadalajara, Mexico; 6 Unidad de Neuro-oncologia, Instituto Nacional de Cancerologia, Mexico City, Mexico; 7 Instituto de Investigaciones Biomédicas, Universidad Nacional Autónoma de México, Mexico City, Mexico; 8 Laboratorio Clínico de Enfermedades Neurodegenerativas, Instituto Nacional de Neurologia y Neurocirugia, "Manuel Velasco Suarez", Mexico City, Mexico; University of South Alabama Mitchell Cancer Institute, UNITED STATES

## Abstract

**Introduction:**

Renin-angiotensin system (RAS) in brain cancer represents a scarcely explored field in neuro-oncology. Recently, some pre- and clinical studies have reported that RAS components play a relevant role in the development and behavior of gliomas. The angiotensinogen (*AGT*) rs5050 genetic variant has been identified as a crucial regulator of the transcription of AGT mRNA, which makes it a logical and promising target of research. The aim of this study was to determine the relationship between the *AGT* rs5050 genetic variant in blood with prognosis in astrocytoma.

**Methods:**

A prospective pilot study was performed on forty-eight astrocytoma patients, who received the standard-of-care treatment. Blood samples were taken prior to surgery and DNA was sequenced using Ion Torrent next-generation sequencing and analyzed by Ion Reporter software. Descriptive, bivariate, multivariate, and survival analyses were performed using SPSS v21, STATA 12 and GraphPad Prism 7.

**Results:**

Median follow-up was 41 months (range 1–48). Survival analysis showed a significant difference between the rs5050 genotypes (p = .05). We found lower survival rates in individuals with the GG-genotype of rs5050 *AGT* compared to patients with the TT- and TG-genotype (2 months vs. 11.5 months, respectively [p = .01]). In bivariate and multivariate analyses, GG-genotype was negatively associated with survival.

**Conclusions:**

In patients with astrocytoma, *AGT* rs5050 GG-genotype was associated with poor prognosis. We propose this germline genetic variant as a complementary biomarker, which can be detected practically and safely in blood samples or saliva.

## Introduction

Gliomas are the most common intrinsic primary tumors of the central nervous system (CNS) in the adult population worldwide, representing approximately 27% of all CNS tumors and 80% of CNS malignant tumors in the United States [1, 2]. Astrocytomas, oligodendrogliomas, and ependymomas are three different glial cell-derived types of gliomas, astrocytomas being the most frequent [2]. Glioblastoma (GBM), the most malignant astrocytic tumor, is considered an incurable disease with a mean survival of 15 months for patients treated with the standard-of-care [3, 4].

The World Health Organization (WHO) grading system belongs to a set of clinical criteria aimed to predict treatment response and outcomes [5]. The WHO classification of tumors of the CNS has become much more accurate with the use of molecular markers, making them an integral part of deciding how to treat gliomas, reducing the interobserver variability and distinguishing new types and variants of tumors [6–8]. Although several biomarkers for diagnosis, risk, and prognosis have been studied, most of them require tumor tissue to be detected [3, 9].

In recent years, biochemical pathways involved in diverse mechanisms, such as the Renin-Angiotensin System (RAS) in blood-pressure control, are now being considered for playing a significant role in carcinogenesis [10]. The RAS, besides its well-known systemic regulation of the circulatory homeostasis, has a local or paracrine function [11]. Local expression of the RAS has been described in multiple tissues, such as liver, kidneys, or pancreas [12]; and also in cancer tissues, such as breast cancer [13], colorectal cancer [14], and renal cell carcinoma [15]. The expression of RAS components have been linked to the hallmarks of cancer [10, 12], and some of those components have been found to be upregulated in some cancer types, including GBM [16–18]. RAS demonstrated involvement in sustaining proliferative signaling, evading growth suppressors, resisting apoptosis, inducing angiogenesis, deregulating cellular energetics, as well as in inflammation, cellular migration, invasion and metastasis [10, 11, 19].

One of the essential RAS components is the human angiotensinogen (*AGT*) gene. The genetic variant rs5050 is a thymidine to guanosine substitution at nucleotide –58 of the 5´UTR of the gene *AGT*. In *in vitro* studies rs5050 has been confirmed to have a functional effect on promoter activity [20]. This has been consistent with studies that have found a correlation between this genetic variant, including haplotypes that contain it, and differences in blood levels of AGT [21, 22]. Additionally, the *AGT* rs5050 has been correlated with an increased risk of developing gastric cancer [23].

Recently, the discovery of RAS peptides and receptors in GBM [17] has urged the planning of clinical studies to elucidate the role of this new concept of the RAS in brain cancer [24–27][28]. A better-characterized analysis of RAS in gliomas has been already described previously [18]. The present study aimed to determine the relationship between the *AGT* rs5050 germline genetic variant with prognosis in astrocytoma.

## Methods

### Source of data

This prospective analytical study and its informed consent were approved by the Institutional Review Board of the National Institute of Neurology and Neurosurgery, Mexico City, Mexico, before recruitment of patients. TRIPOD reporting guideline was implemented [29]. (S1 TRIPOD Checklist).

### Participants

A cohort of adult patients of both sexes, newly diagnosed with primary astrocytoma via histopathology, without prior treatment, were included after signing consent form. Patients with other glial cell-type tumors, prior treatments, or insufficient/degraded DNA samples, were excluded. The included patients underwent surgery for therapeutic and/or diagnostic purpose between 2013 and 2015. After surgery, complementary treatment with standard radiotherapy and chemotherapy were administered to high-grade gliomas and cases with progressive grade II glioma.

### Outcomes

Demographic and clinical information was obtained from medical records by one of the researchers in a blinded fashion. Long-term survival was considered ≥3 years. Patients were grouped according to age into one of four groups for analysis purpose. The performance status was assessed within 1-week before surgery by the Karnofsky performance status (KPS) [30], which is an 11-level scale with scores ranging from normal activity (100) to death (0). A cutoff point of KPS ≥70 was used for analysis. The histological grading was taken from the pathology report, which was based on 2007 WHO classification. The extent of resection (EOR) was evaluated using the postoperative T1-weighted MRI scan with contrast and classified as gross total (100%), subtotal (>90%) or partial (70–90%) resection. The major outcomes were risk and survival. Survival was defined as the lapse of time from when surgery is performed to the patient´s death or last clinic visit.

### Predictors

A sample of 5 mL of blood was taken from each patient before the surgery. Samples were unidentified and labeled using a coding system for internal control, and then, submitted to the laboratory. For DNA genomic extraction, Wizard Genomic DNA Purification Kit (Promega Corporation, Madison, WI, USA) was used. As part of a more extensive study, Ion Torrent NGS (Thermo Fisher Scientific, Waltham, MA, USA) was used for sequencing the genetic variant rs5050. Customized Ion AmpliSeq panel was designed using Ion AmpliSeq designer software. Libraries were constructed using Ion AmpliSeq Library Kit v2.0 according to the manufacturer’s instructions. The library was labeled with an individual adapter given in the Ion Xpress Barcode Adapters Kit. Sequencing was done using Ion 316 chip. Protocols were run on the NGS Ion OneTouch 2 System and the Ion OneTouch ES Instrument according to the user manual. All barcoded specimens were sequenced on the Ion View OT2 Kit. Ion Reporter software was used to perform primary to tertiary analysis, including optimized signal processing, base calling, sequence alignment, and variant analysis. In 12.5% of the samples, orthogonal verification was performed and further verified by conventional Sanger sequencing with 100% validation.

### Sample size and missing data

Sample size was calculated as described by Schoenfeld [31] to determine the minimum sample size for statistical purposes (S1 Table). Complete-case analysis was performed with no missing data identified.

### Statistical analysis methods

In order to compare the allelic and genotypic frequencies of astrocytoma patients in this study, a control group was taken from the 1000 Genomes Browser Phase 3 version 3.7 [32][33][34], where the proportion of the rs5050 *AGT* allele and genotype frequencies were obtained. Hardy-Weinberg equilibrium (HWE) testing was performed on this control group. The wild-type allele and genotype were defined as the most common in the population. We performed descriptive statistics using means, medians, percentages, and maximum and minimum values. The bivariate analysis used contingency tables, and chi-square test and odds ratio were calculated within a 95% confidence interval, and p-value was calculated by Fisher´s exact test. Median follow-up time was calculated using the Schemper and Smith method. The effect of each measured factor on time to death was identified using Kaplan Meier curves, Cox regression models, and Log-rank tests and were used to determine differences in survival function between subgroups. A p-value < .05 of was considered statistically significant. All analyses were performed using SPSS v21 (IBM, Armonk, North Castle, NY), STATA 12 (StataCorp LLC, College Station, TX) and GraphPad Prism 7 (GraphPad Software, Inc., La Jolla, CA).

## Results

### Participants

Forty-eight astrocytoma patients were identified (50% males, 50% females, mean age 49.1, range 22–79 years). The most common tumor location was in the left frontal and temporal lobes, with 16.7% of patients in each location. 68.7% of cases corresponded to high-grade gliomas. Resection of >90% was achieved in 43.7% of patients, and partial resection in 56.3%. Out of the 24 (50%) patients who received chemotherapy, 11 (45.8%) could cover the cost of temozolomide, while the remaining patients received treatment regimes with carboplatin, vincristine, chloroquine, cisplatin, and/or carmustine. Postoperative KPS was ≥70 in 79.2% of patients, and <70 in 20.8%. Additional demographic data is shown in Table 1.

**Table 1:** Demographical and clinical characteristics.

n = 48	n (%)
**Demographic Variables**	
Age*	49 ± 14.1
Sex	M: 24 (50), F: 24 (50)
**Morbidity**	
Hypertension	9 (18.7)
Diabetes Mellitus type 2	8 (16.6)
Smoking	15 (31.2)
Alcohol Abuse	5 (10.4)
Previous Neoplasm	4 (8.3)
**Socio-economic Status**	
Very-Low income	28 (58.3)
Low income	17 (35.4)
Medium income	2 (4.2)
High income	1 (2.1)
**Clinical Variables**	
Age* at Diagnosis	
Grade II	40
Grade III	46.6
Grade IV	54.5
**Tumor Location**	
Frontal	16 (33.3)
Temporal	14 (29.1)
Parietal	9 (18.8)
Occipital	2 (4.2)
Others	7 (14.6)
**Initial Symptom**	
Headache	14 (29.1)
Motor deficit	9 (18.8)
Generalized Seizures	10 (20.8)
Cognitive Functions	8 (16.7)
Partial Seizures	2 (4.2)
Others	5 (10.4)
**Karnofsky Performance Status**	
≥70	38 (79.2)
<70	10 (20.8)
**WHO Grade**	
Grade II	15 (31.3)
Grade III	5 (10.4)
Grade IV	28 (58.3)
**Extent of Resection**	
>90%	21 (43.8)
<90%	27 (56.2)

* = years. M = male, F = female.

### Model development

#### AGT rs5050 gene genetic variant

The rs5050 control group consisted of 2,504 genotypes counted for the worldwide population, including 170 genotypes for the Mexican-ancestry population, which were in HWE after calculations. According to the 1000 Genomes Browser, the distribution of the rs5050 *AGT* alleles and genotypes in the worldwide population is as follows: T-allele was present in 82.4% while G-allele in 17.6%; and the TT-genotype was in 67.9%, TG- in 29% and GG- in 3.1%. In the Mexican-ancestry population in Los Angeles, California, the frequencies were: T-allele in 79.8% and G-allele in 20.2%; and the TT-genotype was in 62.7%, TG- in 34.3% and GG- in 3.0%. From these data, the wild-type and the risk allele and genotype were defined. Then, the proportion of the rs5050 frequencies in our cohort was identified. Out of the 48 patients, the T-allele was present in 77.1% while G-allele in 22.9%; the genotypes corresponded to TT-, TG- and GG-genotypes in 58.3%, 37.5%, and 4.1%, respectively. When comparing genotypic and allelic frequencies, we identified that the allelic and genotypic frequencies of our cohort were similar compared to the Mexican-ancestry population. Additionally, we observed a slightly higher prevalence of the G-allele and GG- and TG-genotypes in our cases in comparison with the worldwide population, which was not statistically significant. Nevertheless, statistical analysis did not reveal *AGT* rs5050 to be a significant risk factor (p = .48 and p = .34) (Table 2).

**Table 2:** *AGT* rs5050 allelic and genotypic frequencies (%).

		(%)	
**Allelic Frequencies (n)**	**T**	**G**	
Present study (96)	77.1	22.9	
MXL (134)*	79.8	20.2	
Global (5,008)*	82.4	17.6	
**Genotypic Frequencies (n)**	**TT**	**TG**	**GG**
Present study (48)	58.3	37.5	4.1
MXL (67)*	62.7	34.3	3.0
Global (2,504)*	67.9	29	3.1

* 1000 Genomes Browser, version 3.7 (*last updated*: *March 1*, *2018*)

MXL = Mexican-ancestry in Los Angeles, California.

### Model specification

#### Survival analysis

Median follow-up was 41 months (range 1–48). The mean survival was 14.8 (range 1–50) months. Long-term survival was reported in five (10.4%) patients. Out of the 40 deceased patients, 17 (42.5%) patients died at ≤0–6 months, 10 (25%) at >6 to ≥12 months, 8 (20%) at >12 to ≤24 months, and 5 (12.5%) at ≥24 months.

**Fig 1:**
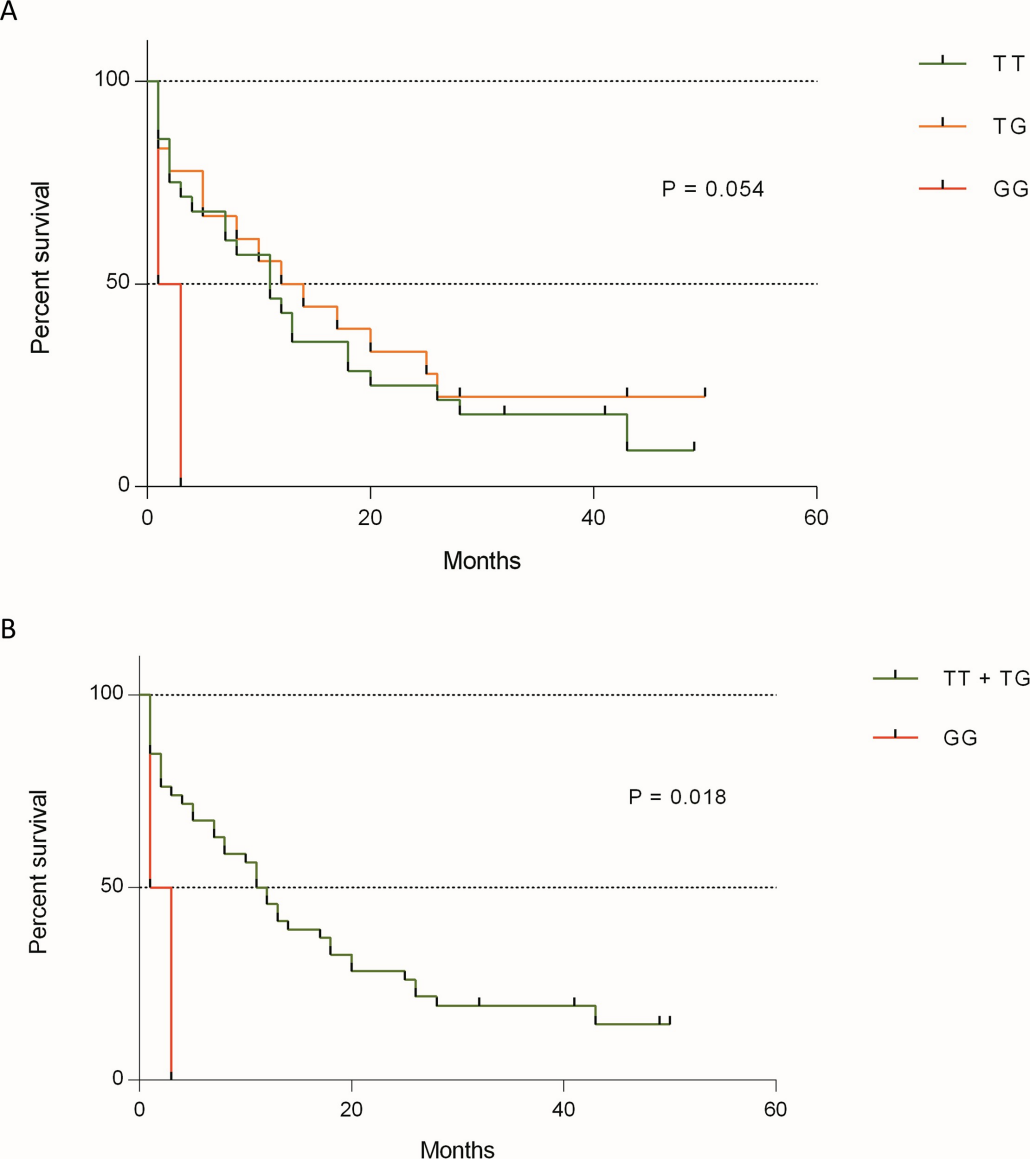
Kaplan-Meier survival estimates. (A) Survival analysis of the *AGT* rs5050 genotypes (TT in green, TG in orange, and GG in red) separately, showing the shorter survival in GG-genotype patients (p = .05). (B) Comparing GG-genotype against TT + TG-genotypes of *AGT* rs5050, patients harboring GG-genotype (in red) exhibited lower survival rates compared to TT + TG (in green) genotypes patients (2 vs. 11.5 months [p = .01]).

The patients were grouped by *AGT* rs5050 genotype, and the survival times were analyzed to obtain the Kaplan-Meier curves. The survival analysis was performed with the rs5050 genotypes (TT, TG, and GG) separately, and with every genotype against the other two genotypes grouped. Survival analysis showed a trend towards significance when genotypes were studied separately (p = .05) (Fig 1A). When comparing each genotype against the two remaining genotypes in Kaplan-Meier survival curves, we found lower survival rates among individuals with the GG-genotype of *AGT* rs5050 (2 months in the GG-genotype carriers vs. 11.5 months in the group of TT- plus TG-genotype patients [p = .01]) (Fig 1B).

Bivariate analysis showed a statistically significant difference between the survival status comparing GG- vs. TG-, and GG- vs. TT-genotypes (p = .011 and p = .016, respectively). Table 3 presents the crude hazard ratio (HR) for survival, by cohort, at the end of the follow-up period. Clinical variables analyses, such as histological grade, KPS, and EOR, among others, were also conducted. Clinical markers of younger age (p = .02) (S1A Fig), lower histological grade (p = .003) (S1B Fig) and higher KPS (p = .04) (S1C Fig) were also related to longer survival.

**Table 3:** Crude hazard ratios for survival.

Cohort	HR	95% CI	*P* value
**Age**			
	1.032	1.01–1.06	0.010
**Gender**			
Female	1.000	-	-
Male	1.514	0.81–2.83	0.193
AGT rs5050 Genotype**			
GG	1.000	-	-
TG	0.130	0.03–0.62	0.011
TT	0.152	0.03–0.70	0.016
Karnofsky Performance Status**			
	0.976	0.96–0.99	0.003
**Extent of Resection**			
>90%	1.000	-	-
<90%	0.718	0.39–1.34	0.296
**Radiotherapy**			
No	1.000	-	-
Yes	0.890	0.48–1.66	0.718
Chemotherapy**			
No	1.000	-	-
Yes	0.858	0.45–1.63	0.639
WHO Grade**			
II	1.000	-	-
III	2.015	0.22–18.26	0.533
IV	4.590	0.62–34.09	0.136
**Smoking**			
No	1.000	-	-
Yes	1.195	0.61–2.33	0.604
**Alcoholism**			
No	1.000	-	-
Yes	1.130	0.40–3.19	0.817
**Familial cancer history**			
No	1.000	-	-
Yes	2.090	1.08–4.07	0.030

** Study cohorts with *p* values < .05 in adjusted hazard ratios calculated using the Cox proportional hazard regression analysis.

### Model performance

In the multivariate Cox proportional HR regression analysis, the model was adjusted for clinical covariables that were considered relevant for survival. After adjusting for gender, KPS, EOR, chemotherapy, radiotherapy, and WHO grade, *AGT* rs5050 genotype remained an independent risk factor for survival with an adjusted HR of 1.000, 0.009 (95% C.I. 0.00–0.09, p = .000) and 0.02 (95% C.I. 0.00–0.17, p = .000), for GG-, TG- and TT-genotypes, respectively. Other covariables that were significant risk factors in this multivariate model for survival were KPS (p = .025), WHO grade (p = .001), and non-chemotherapy (p = 0.000) after adjusting.

## Discussion

In this study, the statistical analysis revealed the GG-genotype of *AGT* rs5050 to be a significantly poor prognosis factor. With a median follow-up of 41 months, GG-genotype carriers of the *AGT* rs5050 presented a statistically significant shorter survival than the other two genotypes (2 vs. 11 months, p = .01). The blood-detected GG-genotype reported this significant correlation with poor prognosis demonstrating value not just as an independent variable but also in the context of other variables. It would give the clinician an additional tool, which combined with other factors, provides a basis for the pursuit of a personalized prognosis for these patients.

### Limitations

Even though the size of the population was relatively small, the number of cases was sufficient for statistical purposes to obtain valid results and to define the direction of further studies. Another limitation of this study is the fact that was performed in a single tertiary referral center, which is entirely dedicated to diseases of the nervous system. Our institution is one of the main hospitals of the Department of Public Health in Mexico, which receives patients from all throughout the central region of the country. The population of this study was composed of Mexicans only. Recently, a US-based study using the zip code tabulation areas codes as a proxy for socioeconomic status (SES), showed that SES did not affect prognosis in patients newly diagnosed GBM [35]. For this reason, the SES of the patients included in this cohort, where almost all of them were classified as very-low and low income, was not considered a bias for survival. However, it is necessary to mention that most of these patients had to cover the cost of their chemotherapy drugs, hindering the treatment with more standardized regimes among the patients. Finally, a reasonable limitation is that O^6^-methylguanine DNA methyltransferase (MGMT) and Isocitrate Dehydrogenase (IDH) mutation were not consistently investigated, as a result of lack of financial resources, and as the patients were recruited before the updated WHO classification was published [36]. Therefore, an attractive opportunity to examine in upcoming studies is the full molecular characterization of the tumor tissue, to analyze the interaction between the expression of *AGT* rs5050 in blood and these biomarkers currently used.

### Interpretation

#### AGT rs5050 gene and angiotensinogen

The *AGT* gene and its genetic variants have been previously studied in the context of the pathogenic mechanisms of essential hypertension, particularly the impact of plasma AGT levels on blood pressure. *In vitro* studies inferred that the transcriptional cis-element of the *AGT* gene regulates blood pressure by managing plasmatic levels of AGT [37]. Yanai *et al*. identified the 5´UTR of the human *AGT* gene as a regulator for the transcription of AGT mRNA [38]. Ishigami *et al*. suggested that rs5050 might influence the level of transcription of AGT mRNA in humans, and therefore modify the plasma AGT concentration. However, they reported a weak correlation between the *AGT* rs5050 and plasmatic AGT levels in a multiple regression analysis, and no differences in plasma AGT concentration among the three genotypes in the analysis of variance [39]. Fourteen years later and using a more sensitive and specific quantification system for human AGT, Balam-Ortiz *et al*. found differences in AGT plasma levels between the genotypes of rs5050, with the GG-genotype associated with the lowest levels. Their results of a bivariate analysis (TT = 25.3±8.3 μg/mL; TG = 22.1±7.1 μg/mL; GG = 19.4±4.8 μg/mL) were statistically significant (TT vs. TG, p = .03; TT vs. GG, p = .05; TT vs. TG+GG, p = .008). Their regression analysis confirmed these differences of the plasmatic AGT levels in the H2 (which contains rs5050) and H8 (which contains –58 wildtype) haplotypes. The H2 haplotype was associated with the lowest plasma AGT levels (–5.1 μg/mL [95% C.I. –8.6 to –1.6], p = .004), while the H8 haplotype was linked to the highest plasma AGT levels (6.5 μg/mL [95% C.I. 2.5–10.6], p = .001) [22].

#### AGT rs5050 and gliomagenesis

The mechanism through which the *AGT* gene influences cancer behavior might stem from genetic variants, bioactive peptides, enzymes, and receptors that have been recently summed to the RAS network [10, 19, 40]. Angiotensin peptides act principally via the AngII receptor type 1 (AT1R) and type 2 (AT2R), and secondarily through the Mas receptor [19, 40]. AT1R and AT2R are receptors with pleiotropic actions with opposing effects. When stimulated, AT1R favors cellular proliferation and angiogenesis while AT2R has antiproliferative attributes [10]. Growing data demonstrate that AT1R is present in several types of neoplasms and that its expression is correlated with tumor growth and a more aggressive disease [12, 41–43]. AT1R stimulates diverse intracellular signaling pathways, leads upregulation of transforming growth factor beta, and induces vascular endothelial growth factor (VEGF) [44–46]. The function of AT2R in cancer is less known. While AT2R has been mainly described as an antiproliferative and proapoptotic mediator, proproliferative and angiogenic effects *in vivo* have also been mentioned in conflicting reports, such as in an AT2R knockout mouse model that showed that the inhibition of AT2R hinders tumor growth by reducing VEGF expression [47].

**Fig 2:**
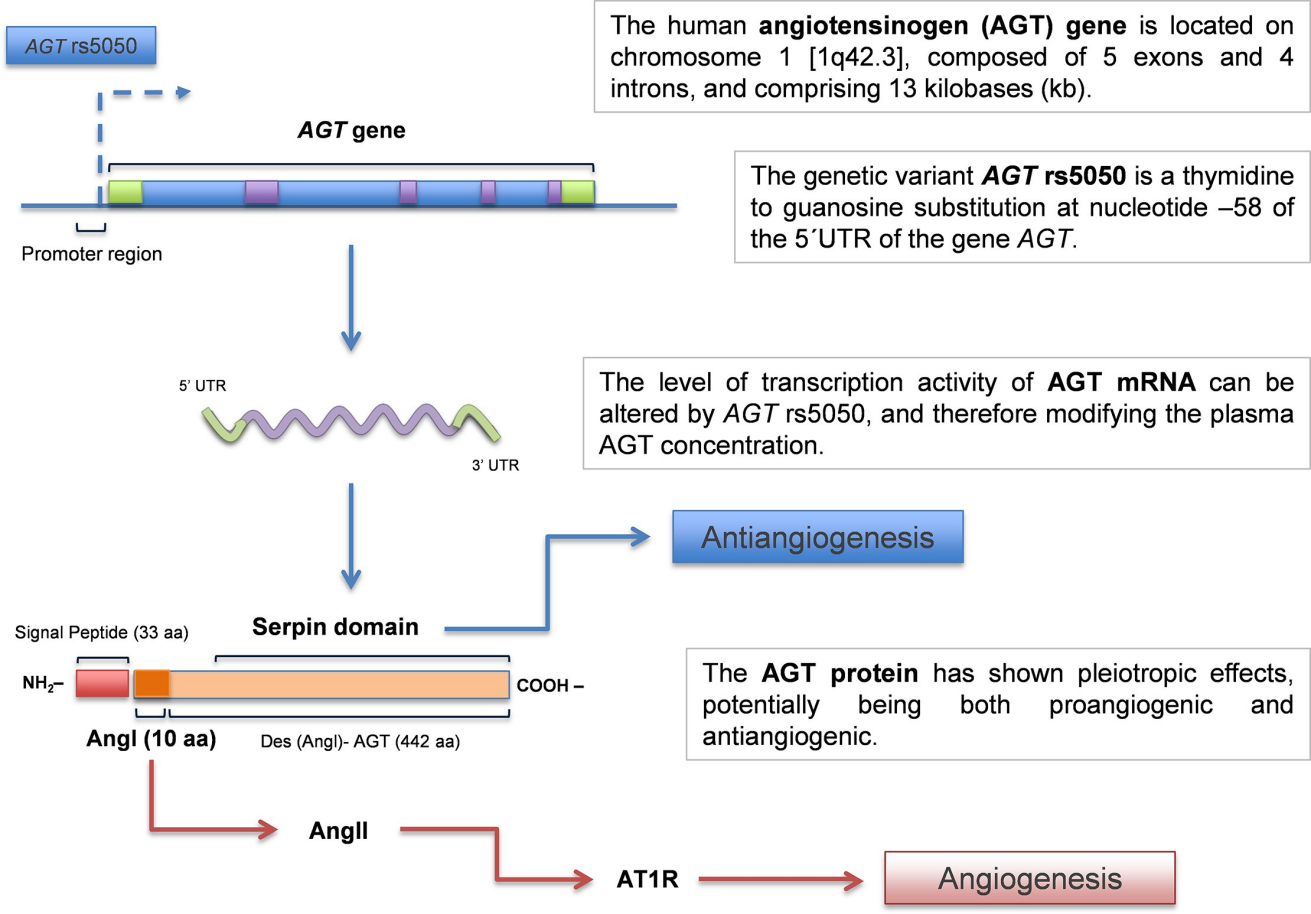
Hypothetical dual mechanism of the *AGT* rs5050 genetic variant. The 5´ upstream core promoter region of the human *AGT* gene, where rs5050 is identified, has been recognized as an authentic regulator for the transcription of AGT mRNA [38]. Differences in the AGT plasma levels were found between the genotypes of rs5050, with the GG-genotype associated with the lowest levels [22]. AGT expresses opposite effects, showing an antiangiogenic property, such as some Serpins family members, and a proangiogenic activity as the precursor of AngII. It might depend upon local conditions that define which of the effects dominates [48].

The promoter activity of *AGT* rs5050 regulates the beginning of the pathway, decreasing the transcription of AGT and its concentration in the plasma. Using well-established *in vitro* and *in vivo* models, Célérier *et al*. demonstrated an antiangiogenic effect of AGT [48]. As they concluded, these opposite effects of AGT, showing an antiangiogenic property as a serpin and a proangiogenic activity as the precursor of AngII, might depend on local conditions that define which of the effects prevails [48] (Fig 2). One of the advantages of the *AGT* rs5050 is that is a germline genetic variant, and thus it can be found in the majority of the patient's cells, including leukocytes from blood and even in normal tissue neighboring neoplasms, unlike the somatic mutations that are found heterogeneously in tumor tissue only [49]. With the knowledge that the GG-genotype is related to decreased plasmatic AGT levels, and that AGT owns a physiological antiangiogenic activity, we infer that the GG-genotype might contribute to a proangiogenic tumor environment, and therefore, to more aggressive behavior and worse outcomes.

#### Implications

To date, just a few clinical studies have explored the feasibility of a relation between RAS components and gliomas [18]. Arrieta *et al*. described the potential of the AT1R as prognosis biomarker [50]. Expression of both receptors, AT1R and AT2R were analyzed in tumor tissue from astrocytoma patients, and a higher prevalence of the two receptors was found in high-grade astrocytomas. AT1R and AT2R were associated with higher cellular proliferation and angiogenesis. AT1R-positive tumors were related to a lower survival rate compared to those which were AT1R-negative [50]. In another study, Lian *et al*. suggested the insertion/deletion (I/D) genetic variant of the *ACE* gene as a risk biomarker for glioma, and performed a case-control study in a Chinese population, which showed that glioma patients had a significantly higher prevalence of the *ACE* DD-genotype detected in blood [51]. The *AGT* rs5050 is so far the only RAS component that is simultaneously related to prognosis and identified by a blood test, which would be both valuable and practical features for a biomarker in a clinical setting. Recent studies have indirectly supported this notion of a relationship between the RAS and prognosis in gliomas, by analyzing the impact of the use of AngII receptor 1 blockers (ARBs) in survival: Carpentier *et al*. described the use of ARBs as a significant prognostic factor for both progression-free survival (PFS) and OS in GBM patients treated with the standard-of-care [25]; and, Levin *et al*. reported an OS benefit offered by ARBs in recurrent GBM patients treated with low-dose Bevacizumab (BVZ) [46]. Recently, Urup *et al*. proposed the *AGT* gene as a predictive biomarker of BVZ response [52]. In that retrospective study, the low expression of *AGT* in the tumor tissue was associated with a prolonged PFS and OS in recurrent GBM patients treated with BVZ [52]. As mentioned, the GG-genotype of rs5050 has been correlated with lower *AGT* expression. Célérier *et al*. [48] reported that AGT could exhibit an antiangiogenic effect as a serpin. Hence, the downregulation of *AGT* might decrease the serpine-mediated antiangiogenic activity of AGT, and consequently, contribute to a proangiogenic tumor microenvironment. This effect might cause the tumor to become more susceptible particularly to anti-angiogenic therapy. This hypothetical mechanism could explain, at least in part, the increased survival seen by Urup *et al*. [52] in those recurrent GBM patients with low expression of AGT when they were treated with BVZ, and also might be the reason for the poor prognosis that was seen in the patients harboring GG-genotype (and not treated with BVZ) in our study. In light of the evidence that the GG-genotype of rs5050 is linked to the lower *AGT* expression, and that the lower *AGT* expression, in turn, is related to longer survival particularly in these GBM subjects with anti-VEGF treatment, we might hypothesize for future studies a plausible role of AGT rs5050 as a potential biomarker of BVZ response.

## Conclusions

The *AGT* r5050 germline genetic variant is proposed as a complementary biomarker to predict survival, which is detected by a blood or saliva test, safely, less invasive, and before surgery. The GG-genotype of *AGT* rs5050 was related to poor prognosis in our cohort, becoming the first study that analyzes and identifies a blood RAS component as a prognosis factor. These results encourage further and broader investigation to endorse this finding and to validate our conclusions in other populations. Future necessary studies regarding *AGT* rs5050 will include its consideration as a treatment-response biomarker or as a druggable target.

## Supporting information

S1 TRIPOD Checklist (DOCX)

S1 Fig Kaplan-Meier survival estimates. Clinical variables analyses, such as histological grade, KPS, and EOR, among others, were also conducted. (A) Younger age (p = .02), (B) lower histological grade (p = .003), and (C) higher KPS (p = .04) were related to longer survival.
(TIF)

S1 Table Parameters used in sample size calculation.
(DOCX)
