# Supplementary figures and images for: Angiotensinogen rs5050 germline genetic variant as potential biomarker of poor prognosis in astrocytoma
Source: PLoS One. 2018 Nov 1;13(11):e0206590. doi: 10.1371/journal.pone.0206590 (PMC6211735; doi:10.1371/journal.pone.0206590)

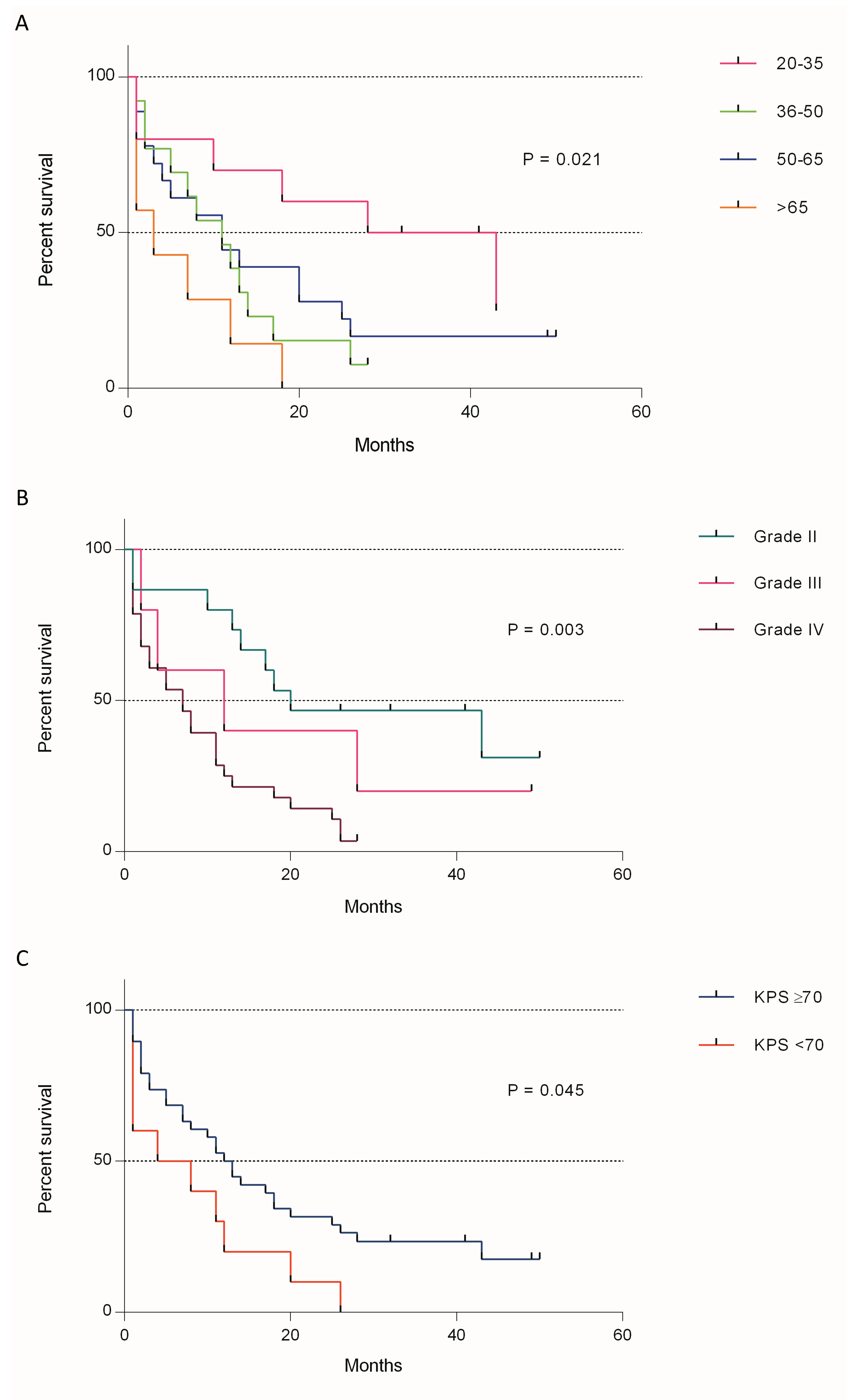

Supplement: S1 Fig — Clinical variables analyses, such as histological grade, KPS, and EOR, among others, were also conducted. (A) Younger age (p = .02), (B) lower histological grade (p = .003), and (C) higher KPS (p = .04) were related to longer survival. (TIF) [file pone.0206590.s002.tif]
