# Supplementary material for: Angiotensinogen rs5050 germline genetic variant as potential biomarker of poor prognosis in astrocytoma
Source: PLoS One. 2018 Nov 1;13(11):e0206590. doi: 10.1371/journal.pone.0206590 (PMC6211735; doi:10.1371/journal.pone.0206590)
Supplement: S1 Table — Parameters used in sample size calculation. (DOCX) [file pone.0206590.s003.docx]

| **Supplementary Table 1.** | |
| --- | --- |
| **Parameters used to calculate the Minimum Sample Size** |  |
| Relative Risk to detect | 2.9 |
| Proportion exposed | 0.20^*^ |
| Proportion of censured observations | 0 |
| Confidence level | 0.95 |
| Statistical power | 0.8 |
| Correlation with the other factors included in the model | 0.28 |
| **Minimum Sample Size** | 47 |
| **Proportion of individuals with G allele (%)* |  |
